# Modern Sociology

**Published:** 1899-05-13

**Authors:** 


### Modern Sociology.

### THE POOR OF DENMARK.

Denmark is one of the countries which have accepted the principle of pensioning the aged poor, without asking them to contribute in their youth to their future maintenance. The assumption is that everyone who has attained the age of sixty years, without, during that time, obtaining help from the poor-law, has contributed to the State more than he has received from it, and therefore has a claim to be supported during his declining days. It may be doubted if the principle is altogether sound. The tramp who had during his wandering career managed to keep clear of the casual ward might, on the face of it, claim to have been a self-supporting citizen, when on his sixtieth birthday he applied for his pension. But, as a matter of fact, the poor-law system of Denmark divides the poor into so many more classes than we do here that it is unlikely so much harm is done as, from a British point of view, would seem to be inevitable. The poor-law of Denmark throughout makes a distinction between the respectable and the disreputable poor. Separate poorhouses are provided for the two classes. Out-relief does not meet with the same condemnation in Denmark that attends it here. On the contrary, such relief is rather the rule than the exception; and it is only those persons who cannot be relieved at their own homes who are sent to the poorhouse. Moreover, the occupants of the poorhouse are not always absolutely destitute; they may pay in part, or even wholly, for their maintenance. In short, the Danish poorhouse has no necessary connection with pauperism, but rather with loneliness, or other circumstances which render it inadvisable for a poor person to remain at home. The poorhouses are in themselves very small, seldom accommodating more than eight or ten inmates. They thus bear no comparison with English workhouses. A further development, which, amongst us, is barely yet even in the experimental stage, is the "poor-farm," on which pauper labour is employed. A poor-farm, of which there are 270 in the country, contains about forty inmates. These do all the work of the farm, and sometimes their labour is hired out to farmers. Knowing what the economic results of such a measure were in our own country, we are rather doubtful if this plan is good for the people of Denmark; but the underlying problems of economics do not seem to trouble the legislators of that country. One wise provision there certainly is in the Danish poor-law, namely, that the receipt of poor-law relief not only disfranchises the pauper, but prevents his marrying for five years after, from the date of the receipt of relief, except with the consent of the parish authorities. A woman, however, does not need to ask this permission. Whether the refusal of the permission to contract a legal marriage really secures its object may be doubted, even though the authorities are supposed to have the power also to prohibit irregular unions. According to the latest available statistics, the average illegitimate births was from 9 to 11 per cent. in the country districts, and from 19 to 22 per cent. in Copenhagen. Whether this surprising number of illegitimate births is due to a traditional laxity of morals cannot be clearly known; but it is evident that the powers of the poor-law authorities to prevent irregular unions has not done much to improve it.

While in some ways the path of the pauper seems to be made so easy in Denmark, there is another side to the question. The authorities there have the right to deal in very severe fashion with those among the poor whom they consider disreputable. There are workhouses—quite different from the poorhouses—to which refractory paupers may be sent. These are really houses of correction, where the offender may be confined for a period not exceeding six months. Moreover, indoor paupers are divided into grades, according to their deserts, and separate institutions are assigned to the various classes. Thus the vagrant when he retires to the workhouse to end his days, is not placed in the same house, nor does he enjoy the same treatment as the deserving poor. It is admitted, however, by many officials, that there is a tendency to be too easy in giving relief. Under one head or another every applicant can claim help, and under these circumstances officials are not too careful about the grading of them. Still, it is claimed that there is little hereditary pauperism in Denmark. Pauper children are almost universally boarded out; and often they do not know that they are paupers. Herein, of course, lies the great advantage of boarding-out. But we should think that under the Danish system there would be no need for hereditary leanings to make the path to pauperism easy.

Of the aged pensioners, not to be regarded as paupers, some are housed in institutions, but the majority receive out-relief. The allowance given varies from 50 to 200 kroner (it takes a little over 18 kroner to make £1). More than half the recipients are widows and single women, who everywhere find it difficult to make a living in old age. The pensioners are divided into four classes, the lowest is that of unskilled labour; the second includes artisans, small tradespeople, and the like; the third, traders, peasant proprietors, &c.; the fourth, teachers, clerks, and the professional classes generally. These form a very small proportion of the whole. In fact, 80 per cent. of the recipients come from the two first classes, the second being slightly the larger. This may be due partly to the fact that so many of the occupations followed by women, such as nurses, are included in the second class. The cost of old-age pensions is slightly larger than any of the other items into which the expense of poor-relief is divided. In 1895 it amounted to 688,941 kroner. Since the pension law came into operation in 1892 the amount thus expended has steadily gone up, but that required for poor-relief, apart from hospital treatment, has as regularly gone down. Presumably a smaller number of the deserving poor are now classed as paupers.

There are no poor-law hospitals in Denmark. Sick paupers are treated in the ordinary hospitals, and the parish or "commune" to which they belong pays for them. A very useful institution in Denmark is the sick club, to which a great many of the people belong, which secures for its members the right to be treated in the general hospitals at a reduced fee. A large number of the people belong to these clubs.

It is evident that the relief of the poor is conducted on other lines in Denmark from what it is here. Whether we could with safety adopt any of the fashions in vogue there is doubtful. A small, sparsely-peopled agricultural country like Denmark presents few of the problems that face us in Britain; and the principle on which relief is administered seems to imply a personal acquaintance on the part of the guardians or relieving officers with the applicants, which, at least in our large cities, is practically unattainable.

